# In Vitro Antibacterial Activity of a Novel Resin-Based Pulp Capping Material Containing the Quaternary Ammonium Salt MAE-DB and Portland Cement

**DOI:** 10.1371/journal.pone.0112549

**Published:** 2014-11-12

**Authors:** Yanwei Yang, Li Huang, Yan Dong, Hongchen Zhang, Wei Zhou, Jinghao Ban, Jingjing Wei, Yan Liu, Jing Gao, Jihua Chen

**Affiliations:** 1 State Key Laboratory of Military Stomatology, Department of Prosthodontics, School of Stomatology, Fourth Military Medical University, Xi'an, China; 2 State Key Laboratory of Military Stomatology, Department of General Dentistry and Emergency, School of Stomatology, Fourth Military Medical University, Xi'an, China; 3 Department of Clinical Nursing, School of Nursing, Fourth Military Medical University, Xi'an, China; LSU Health Sciences Center School of Dentistry, United States of America

## Abstract

**Background:**

Vital pulp preservation in the treatment of deep caries is challenging due to bacterial infection. The objectives of this study were to synthesize a novel, light-cured composite material containing bioactive calcium-silicate (Portland cement, PC) and the antimicrobial quaternary ammonium salt monomer 2-methacryloxylethyl dodecyl methyl ammonium bromide (MAE-DB) and to evaluate its effects on *Streptococcus mutans* growth in vitro.

**Methods:**

The experimental material was prepared from a 2∶1 ratio of PC mixed with a resin of 2-hydroxyethylmethacrylate, bisphenol glycerolate dimethacrylate, and triethylene glycol dimethacrylate (4∶3∶1) containing 5 wt% MAE-DB. Cured resin containing 5% MAE-DB without PC served as the positive control material, and resin without MAE-DB or PC served as the negative control material. Mineral trioxide aggregate (MTA) and calcium hydroxide (Dycal) served as commercial controls. *S. mutans* biofilm formation on material surfaces and growth in the culture medium were tested according to colony-forming units (CFUs) and metabolic activity after 24 h incubation over freshly prepared samples or samples aged in water for 6 months. Biofilm formation was also assessed by Live/Dead staining and scanning electron microscopy.

**Results:**

*S. mutans* biofilm formation on the experimental material was significantly inhibited, with CFU counts, metabolic activity, viability staining, and morphology similar to those of biofilms on the positive control material. None of the materials affected bacterial growth in solution. Contact-inhibition of biofilm formation was retained by the aged experimental material. Significant biofilm formation was observed on MTA and Dycal.

**Conclusion:**

The synthesized material containing HEMA-BisGMA-TEGDMA resin with MAE-DB as the antimicrobial agent and PC to support mineralized tissue formation inhibited *S. mutans* biofilm formation even after aging in water for 6 months, but had no inhibitory effect on bacteria in solution. Therefore, this material shows promise as a pulp capping material for vital pulp preservation in the treatment of deep caries.

## Introduction

Pulpal vitality is critical to the maintenance of the structural integrity and normal physiological function of teeth. As our understanding of the importance of pulp in tooth health increases, methods for preserving pulp vitality during caries treatment even after exposure during caries removal are in great demand [1]. Currently, pulp capping is the primary method for preserving vital pulp, but the success rate of this approach during the treatment of deep caries is low at only 33% [2]. The presence of bacteria is the major reason for failure [3]. Bacteria located in deep caries can induce severe inflammatory reactions in the pulp and even cause pulp necrosis [4]. Therefore, the prevention of bacterial infections is an important objective for improving pulp capping methods in the treatment of deep caries.

In general, an ideal pulp capping material should possess both excellent antibacterial properties and the ability to induce mineralized tissue formation [5]. Currently, the most common pulp capping materials used clinically include various formulations of calcium hydroxide [Ca(OH)_2_] and mineral trioxide aggregate (MTA). MTA has been shown to induce less pulp inflammation and greater dentin bridge formation as well as offer superior structural qualities compared to Ca(OH)_2_ [6]–[8]. The better performance of MTA compared to Ca(OH)_2_ may be due to continued dissolution of Ca(OH)_2_ paste, which has a prolonged irritant effect (release of basic ions) on pulp tissues [6]. Although both Ca(OH)_2_ and MTA promote the formation of mineralized tissue, they lack good antibacterial properties [9]–[11], and thus, cannot prevent the bacterial infection that commonly leads to treatment failure in cases of deep caries. Therefore, a novel pulp capping material that offers a combination of excellent antibacterial properties and the ability to induce mineralized tissue formation is highly desired.

Dental resins modified with a quaternary ammonium salt (QAS) have been shown to have excellent antibacterial properties [12]–[19]. QAS monomers such as 12-methacryloyloxydodecylpyridinium bromide (MDPB) and other antibacterial monomers can be copolymerized with dental resins to form antibacterial polymer matrices that effectively inhibit bacterial growth [12]–[19]. Our research group developed a novel QAS monomer, 2-methacryloxylethyl dodecyl methyl ammonium bromide (MAE-DB), which contains two polymerizable methacrylate groups that facilitate its facile polymerization with dental resin monomers and other MAE-DB monomers [20]. MAE-DB exhibits strong bactericidal action against oral bacteria [20] and can be copolymerized with dental resin monomers to form an antibacterial composite resin that effectively inhibits bacterial growth even after a 6-month aging process [21].

The ability of MTA to induce mineralized tissue formation has been attributed to its components [6]–[8], [22]. According to the manufacturer, MTA is formed by mechanically mixing three powder ingredients: Portland cement (PC, 75%), bismuth oxide (20%), and gypsum (5%) [23], [24]. Thus, PC is the major component of MTA [24], [25], and several studies have demonstrated that PC shares the same physical and chemical properties with MTA [26]–[28] as well as the same antimicrobial activity [11], biocompatibility [29], [30], and pulp capping effectiveness [22].

To create a novel pulp capping material that can both prevent bacterial infection and support mineralized tissue formation, we synthesized a composite resin containing MAE-DB and PC. We then investigated the immediate and long-term antibacterial effects of this new light-cured pulp capping material against *Streptococcus mutans* in vitro.

## Materials and Methods

### Specimen preparation and aging treatment

**Figure 1:**
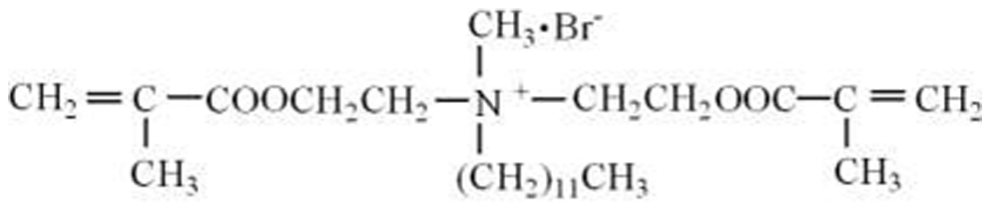
Structure of the QAS monomer MAE-DB.

The structure of the QAS monomer MAE-DB is presented in Figure 1. The resin matrix of the experimental light-curable material evaluated in this study was composed primarily of 2-hydroxyethylmethacrylate (HEMA, Sigma–Aldrich, St. Louis, MO, USA), bisphenol glycerolate dimethacrylate (BisGMA, Esstech, Essington, PA, USA), and triethylene glycol dimethacrylate (TEGDMA, Esstech) with a mass ratio of 4∶3∶1 (Table 1). The photoinitiator camphorquinone (CQ, Sigma-Aldrich) and coinitiator ethyl 4-(dimethylamino)benzoate (EDMAB, Sigma-Aldrich) were added at concentrations of 0.5 wt% of the resin matrix each. The compositions of the experimental (HEMA-BisGMA-TEGDMA resin with MAE-DB and PC), positive control (HEMA-BisGMA-TEGDMA resin with MAE-DB only), and negative control (HEMA-BisGMA-TEGDMA resin without MAE-DB or PC) materials are listed in Table 1. In the experimental material, MAE-DB monomer was added as an immobilized bactericide at 5 wt% in the HEMA-BisGMA-TEGDMA resin, and white PC (P. W. 52.5, Aalborg, Anqing, China) was then added at a PC:HEMA-BisGMA-TEGDMA resin mass ratio of 2∶1. HEMA-BisGMA-TEGDMA resin containing 5 wt% MAE-DB without PC served as the positive control. HEMA-BisGMA-TEGDMA resin without MAE-DB or PC served as the negative control. Two common pulp capping materials, white ProRoot MTA (Dentsply, Tulsa, OK, USA) and Dycal (Dentsply, Milford, DE, USA), were used for comparisons to commercially available materials.

**Table 1:** Compositions of experimental resin composites.

Group	HEMA-BisGMA-TEGDMA %	CQ %	EDMAB %	MAE-DB %	Portland cement %
Negative control	99	0.5	0.5	0	0
Positive control	94	0.5	0.5	5	0
Experimental material	31.33	0.17	0.17	1.67	66.7

Data are given in mass percentages.

HEMA-BisGMA-TEGDMA refers to a HEMA, BisGMA, and TEGDMA mixture at a 4∶3∶1 ratio.

Note that 5% MAE-DB in HEMA-BisGMA-TEGDMA resin equals 1.67% of the total content upon the addition of PC.

For sample preparation, the experimental, positive control, and negative control materials were placed into disk-shaped organic glass molds (inner diameter of 10 mm and depth of 1.5 mm). The top and bottom surfaces were covered with a Mylar strip and a microscope slide, which was slightly pressed to remove excess material. The resins were photo-cured for 60 s on each side with a light activation unit (QHL75, Dentsply). After the resin disks were removed from the mold, they were submersed in distilled water with agitation for 1 h for the removal of any uncured monomer [31]. According to the manufacturer's instructions, MTA was mixed with sterile water (powder:liquid ratio of 3∶1), and Dycal was prepared by mixing equal amounts of catalyst paste and base paste. The same organic glass molds and procedures (except for light irradiation) were used to prepare disks of these materials. All of the prepared disks were sterilized with ethylene oxide, followed by degassing in the fuming cupboard for more than 48 h before testing [17], [31].

For aging, specimens of each group were placed in wells of a 24-well plate containing 1 ml deionized water, which was changed every 48 h. After aging for 6 months at 37°C, specimens were retrieved, sterilized, and subjected to the following experiments [17].

### Bacterial strain and culture conditions

*Streptococcus mutans* UA159 (State Key Laboratory of Military Stomatology, School of Stomatology, Fourth Military Medical University, Xi'an, China) was cultured overnight at 37°C in brain-heart infusion (BHI) broth (Difco, Detroit, MI, USA) in an anaerobic atmosphere enriched with 5% CO_2_. The resulting bacterial suspension was adjusted to an optical density (OD) of 0.5 at 600 nm and then diluted 1∶100 with fresh BHI for further use [31].

### Bacterial growth on material surfaces and in culture medium

The sterile disks prepared for testing with or without aging were placed in wells of a 24-well plate with 2 ml BHI broth. Then 20 µl of the diluted *S. mutans* suspension was added to each well. After 24 h in anaerobic culture, biofilm formation on the disk and the planktonic bacteria in the culture medium were assessed using the following experimental techniques [31].

The total number of viable bacteria was evaluated according to the number of colony-forming units (CFU) both on the disk surface and in the culture medium over each disk. After bacteria within biofilms are properly dispersed and diluted, each viable bacterium results in a single, countable colony on an agar plate. After 24 h in culture to allow biofilm growth, disks were washed twice with PBS and then transferred into tubes (15-ml sterile centrifuge tubes, Nest, China) with 2 ml fresh BHI. Biofilms on individual disks were harvested by sonication (3510R, Branson, Danbury, CT, USA) for 3 min and vortex mixing at maximum speed for 20 s using a vortex mixer (Fisher Scientific, Pittsburgh, PA, USA), for removing and dispersing the bacteria [19].

Once the disks had been removed from the wells for biofilm harvesting, planktonic bacteria in the original medium samples were mixed thoroughly by repeated pipetting to achieve a homogeneous bacterial suspension. The bacterial suspensions from both the biofilms on the disks and the planktonic bacteria in the medium were serially diluted, spread onto BHI agar plates, and incubated for 1 day at 5% CO_2_ and 37°C for CFU analysis (n = 6), following previously reported methods [19], [31].

### Bacterial metabolic activity on material surfaces and in culture medium

The bacterial suspensions obtained from biofilms formed on the disks and planktonic bacteria in the medium were prepared as described in Section 2.3. After brief mixing via repeated pipetting, 200-µl aliquots of the bacterial suspensions were transferred to wells of a 96-well plate, and then 20 µl of Cell Counting Kit-8 (CCK-8) dye solution was added to each well and incubated at 37°C in 5% CO_2_ for 2 h. Instead of 3-(4,5-dimethylthiazol-2-yl)-2,5-diphenyltetrazolium bromide (known as MTT), the CCK-8 assay uses WST-8(2-(2-methoxy-4-nitrophenyl)-3-(4-nitrophenyl)-5-(2,4-disulfophenyl)-2H-tetrazolium, monosodium salt, which produces a yellow, water-soluble formazan production upon reduction by dehydrogenases in metabolically active bacteria. The absorbance at 450 nm of the resulting solution in each well was measured using a microplate reader (SpectraMax M5, Molecular Devices, Sunnyvale, CA). Each sample was assayed in triplicate, and an average value was calculated for each sample. A higher absorbance value indicates a higher formazan concentration, which in turn indicates the presence of more metabolically active bacteria in the sample.

### Live/dead staining for visualization of *S. mutans* viability on material surfaces

Biofilm formation on sample disks during 24 h in culture was achieved as described in Section 2.3. The disks coated with biofilms were washed three times with sterile saline to remove loose bacteria, and then the remaining bacteria were stained using the Live/Dead BacLight Bacterial Viability Kit L13152 (Molecular Probes, Invitrogen, Eugene, OR, USA) with a 15-min incubation in the dark at room temperature. With this staining kit, live bacteria produce green fluorescence upon staining with Syto 9, and bacteria with compromised membranes produce red fluorescence upon staining with propidium iodide [21], [32]. After incubation with the fluorescent dyes, the samples were rinsed gently with distilled water and observed by confocal laser scanning microscopy (CLSM, FluoView FV1000, Olympus, Tokyo, Japan). Excitation with a 488-nm laser revealed the green fluorescence emission of live bacteria, and excitation with a 543-nm laser revealed the red fluorescence emission of bacteria with damaged membranes [21]. Three disks were used for each condition (type of material and aging status). Four images were collected at random locations on each disk, yielding 12 images per condition.

### Scanning electron microscopy (SEM) of *S. mutans* on the tested material surfaces

Biofilm formation on sample disks during 24 h in culture was achieved as described in Section 2.3. Then the disks coated with biofilms were gently rinsed with PBS, soaked in 3% glutaraldehyde at 4°C overnight, washed twice with PBS, dehydrated in a graded series of ethanol solutions, and then dried in a critical-point drier [21]. After sputter coating of the samples with gold using an ion sputter (JFC-1100E, JEOL, Tokyo, Japan), all specimens were observed by field emission SEM (FESEM; S-4800; Hitachi Ltd, Tokyo, Japan).

### Statistical analysis

One-way and two-way analyses of variance (ANOVAs) were performed to detect the significant effects of the variables (material type and aging status) on CFU count and bacterial metabolic activity. Tamhane multiple comparison test was used to compare differences between any two groups, with significance assumed at a p-value of 0.05. Standard deviation (SD) values serve as estimates for the standard uncertainty associated with particular measurements.

## Results

### CFU counts of *S. mutans* on the surfaces of the tested materials and in the culture medium away from the surfaces

Table 2 shows CFU counts of *S. mutans* on the surfaces of the tested materials with different aging treatments. Two-way ANOVA showed that only material type had significant effect on the CFU count (*P*<0.05). Differences among all the subgroups for both fresh and aged materials were assessed by one-way ANOVA. For each material, aging had no significant effect on the CFU count (*P*>0.05). The numbers of CFUs from *S. mutans* biofilms on the experimental material and positive control material were significantly less by about an order of magnitude than that for the negative control material (*P*<0.05). No significant difference was observed between the experimental and positive control groups (*P*>0.05). In contrast, the CFU counts from *S. mutans* biofilms on MTA and Dycal were significantly greater by about one order of magnitude compared to that for the negative control material (*P*<0.05) and by about two orders of magnitude compared to those for the experimental and positive control materials (*P*<0.05 for both). No significant difference was found between MTA and Dycal (*P*>0.05). Together these results indicate that the addition of PC at a 2∶1 mass ratio to HEMA-BisGMA-TEGDMA resin containing 5% MAE-DB did not diminish the ability of the antibacterial resin to inhibit *S. mutans* growth on its surface even after 6 months of aging. Conversely, the growth of *S. mutans* on the surfaces of MTA and Dycal was even greater than that on HEMA-BisGMA-TEGDMA resin without MAE-DB or PC.

**Table 2:** CFU counts from *S. mutans* biofilms on material surfaces.

Material	Biofilm CFU (per disk)	
	Without aging	With aging
Negative control	5.95(0.98) ×10^7A^	6.12(1.01)×10^7A^
Positive control	3.53(0.91)×10^6B^	3.72(0.86)×10^6B^
Experimental material	6.14(1.13)×10^6B^	6.27(1.02)×10^6B^
MTA	4.71(0.76)×10^8C^	4.64(0.81)×10^8C^
Dycal	4.55(0.88)×10^8C^	4.65(0.97)×10^8C^

CFU values represent the mean (SD) of six replicates, and data were analyzed with one-way and two-way ANOVA at a significance level of 0.05. Values with dissimilar superscript letters are significantly different from each other (p<0.05). Values with the same superscript letter are not significantly different (p>0.05).

Table 3 lists the CFU counts of *S. mutans* from the culture medium away from the surfaces of the tested materials with different aging conditions. Two-way ANOVA showed that both material type and aging had no significant effect on the CFU count (all *P*>0.05). In addition, one-way ANOVA revealed no significant differences among all subgroups with different aging conditions (all *P*>0.05). These results indicate that the *S. mutans* growth in the culture medium was not inhibited by the experimental material, the positive control material, or the two commercial materials in comparison to that in the medium away from the negative control material.

**Table 3:** CFU counts from *S. mutans* in culture medium away from the material surfaces.

Medium sample	*S. mutans* CFU (per mL)	
	Without aging	With aging
Culture medium of negative control	1.52(0.16)×10^9A^	1.57(0.13)×10^9A^
Culture medium of positive control	1.50(0.28)×10^9A^	1.43(0.20)×10^9A^
Culture medium of experimental material	1.53(0.21)×10^9A^	1.61(0.19)×10^9A^
Culture medium of MTA	1.41(0.23)×10^9A^	1.49(0.16)×10^9A^
Culture medium of Dycal	1.42(0.23)×10^9A^	1.51(0.19)×10^9A^

CFU values represent the mean (SD) of six replicates, and data were analyzed with one-way and two-way ANOVA at a significance level of 0.05. Values with the same superscript letter are not significantly different (p>0.05).

### Metabolic activity of *S. mutans* on the surfaces of the tested materials and in the culture medium away from the surfaces

**Figure 2:**
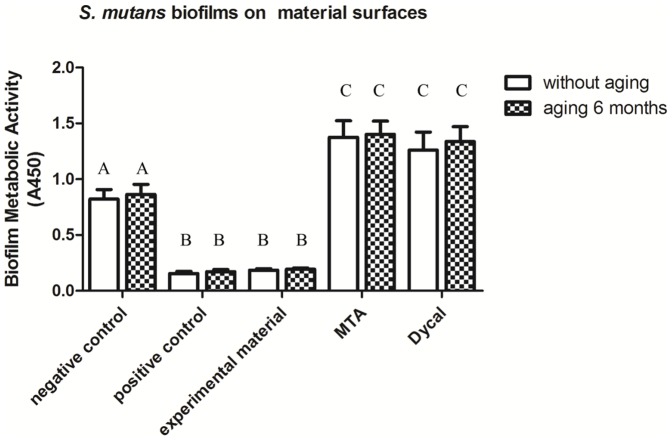
Metabolic activity of *S. mutans* in biofilms on material surfaces. Metabolic activity of *S. mutans* in biofilms on material surfaces (mean±SD; n = 6) for negative control, positive control, and experimental materials as well as MTA, Dycal, and the corresponding aged samples for each material. Absorbance values were analyzed with one-way and two-way ANOVA at a significance level of 0.05. Values with dissimilar letters are significantly different from each other (p<0.05). Values with the same letter are not significantly different (p>0.05).

The metabolic activity data for *S. mutans* biofilms on the material surfaces are plotted in Figure 2. Two-way ANOVA showed that only material type had a significant effect on the metabolic activity of the bacteria (*P*<0.05). According to one-way ANOVA, for each material, aging had no significant effect on the metabolic activity (*P*>0.05 for all materials). Regardless of the aging condition, the greatest absorbance values and thus highest levels of metabolic activity were observed for *S. mutans* biofilms formed on MTA and Dycal (*P*<0.05 compared to all other material types). Conversely, the lowest levels of metabolic activity were observed for *S. mutans* biofilms on the experimental and positive control materials, indicating these materials had the strongest antibacterial activity (*P*<0.05 compared to all other material types). These results are consistent with those for bacterial growth in *S. mutans* biofilms on the tested material surfaces based on CFU counts.

**Figure 3:**
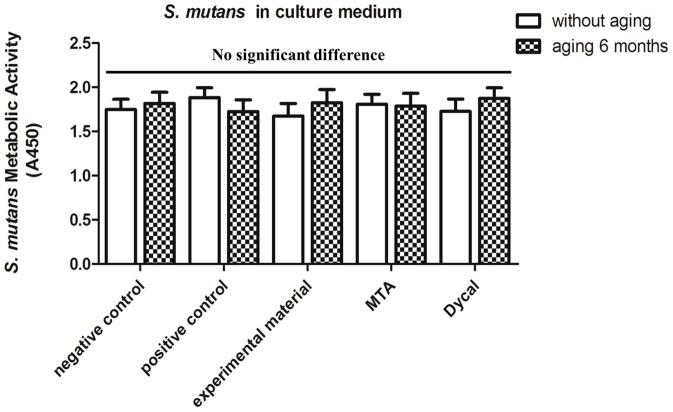
Metabolic activity of *S. mutans* in culture medium away from the surfaces. Metabolic activity of *S. mutans* in culture medium over the surfaces (mean±SD; n = 6) of the negative control, positive control, and experimental materials as well as MTA, Dycal, and the corresponding aged samples for each material. Absorbance values were analyzed with one-way and two-way ANOVA at a significance level of 0.05, and no significant differences were observed between any conditions (p>0.05).

The metabolic activity data for *S. mutans* in the culture medium away from the disk samples are plotted in Figure 3. Two-way ANOVA showed that neither material type nor aging condition significantly affected the metabolic activity of bacteria in the culture medium (all *P*>0.05). In addition, one-way ANOVA revealed the lack of significant differences in *S. mutans* metabolic activity in the culture medium among all material types and aging conditions (all *P*>0.05). These results also are consistent with those for bacterial growth in *S. mutans* in the culture medium over the surfaces based on CFU counts.

### Viability of *S. mutans* on the tested material surfaces

**Figure 4:**
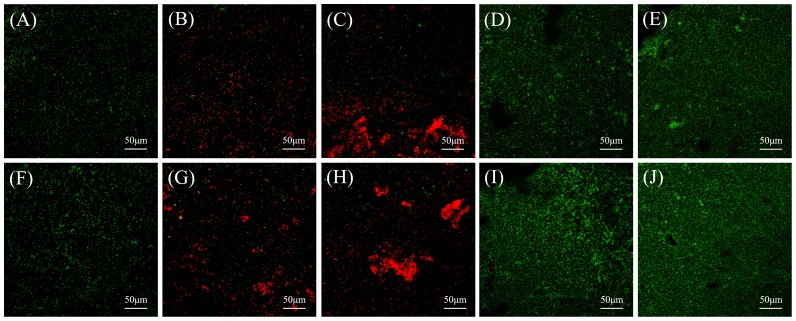
Representative CLSM images of Live/Dead-stained biofilms on material surfaces. Representative CLSM images of Live/Dead-stained biofilms after 24 h of anaerobic growth on the tested material surfaces: (A) negative control material, (B) positive control material, (C) experimental material, (D) MTA, and (E) Dycal. Biofilms on the corresponding aged samples are shown in (F)–(J). Live bacteria exhibited green fluorescence, and bacteria with compromised membranes exhibited red fluorescence. Scale bars, 50 µm.

Figure 4 shows representative CLSM images of Live/Dead-stained *S. mutans* biofilms after 24 h of anaerobic growth on the material surfaces. Both fresh and aged negative control surfaces (Fig. 4A and F) were covered with primarily live bacteria. By contrast, both fresh and aged positive control surfaces (Fig. 4B and G) showed more dead bacteria, compared to the negative control surfaces. Compared to that on the control surfaces, the total amount of bacteria was greatly increased on both fresh and aged experimental surfaces (Fig. 4C and H), and these surfaces were covered primarily with dead bacteria. The amounts of bacteria on fresh and aged MTA surfaces (Fig. 4D and I) also were greater than on control surfaces, but the MTA surfaces were covered primarily with live bacteria. The staining results on fresh and aged Dycal surfaces (Fig. 4E and J) were qualitatively similar to those on MTA surfaces. For each material, the Live/Dead staining results were qualitatively similar between the fresh and aged surfaces. These results are consistent with those for *S. mutans* growth according to CFU count and metabolic activity in biofilms on the tested material surfaces.

### SEM imaging of *S. mutans* on the tested material surfaces

**Figure 5:**
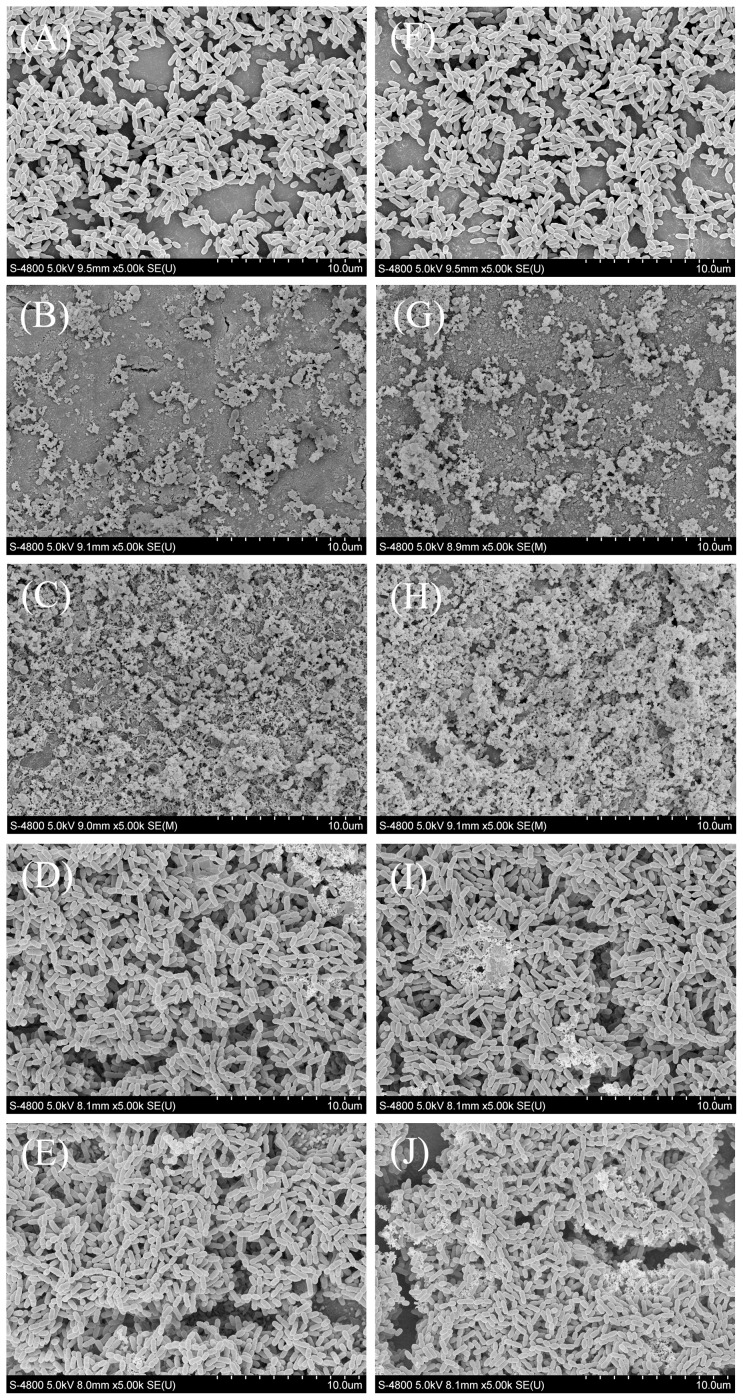
Representative SEM images of *S. mutans* biofilms on material surfaces. Representative SEM images of *S. mutans* biofilms after 24 h of anaerobic growth on the tested material surfaces: (A) negative control material, (B) positive control material, (C) experimental material, (D) MTA, and (E) Dycal. Biofilms on the corresponding aged samples are shown in (F)–(J).

Figure 5 shows representative SEM images of *S. mutans* biofilms after 24 h of anaerobic growth on the tested material surfaces. Both fresh and aged negative control surfaces (A and F) were covered primarily with live bacteria with intact membranes. Both fresh and aged positive control surfaces (B and G) were covered with considerably more dead bacteria with compromised membranes, compared to the negative control surfaces. The total amounts of bacteria on both fresh and aged experimental surfaces (C and H) were greater than those on the control surfaces, and the experimental surfaces were covered primarily with dead bacteria. The total amounts of bacteria on fresh and aged MTA surfaces (D and I) also were greater than those on control surfaces, but the MTA surfaces were covered primarily with live bacteria. The total amount and viability of bacteria on fresh and aged Dycal surfaces according to SEM imaging (E and J) were qualitatively similar to those on MTA surfaces. For each material, the SEM observations were qualitatively similar between fresh and aged samples. These results for *S. mutans* biofilms on the tested material surfaces are consistent with those obtained by counting CFUs, measuring metabolic activity, and labeling cells with the Live/Dead fluorescent staining kit.

## Discussion

*S. mutans* is a major pathogen causing human dental caries [21] and was therefore chosen for evaluation of the antibacterial effects of the materials prepared in this study. The present study investigated the immediate and long-term antibacterial activity of a novel light-cured pulp capping composite resin containing PC filler and MAE-DB monomer on *S. mutans* both on its surface and in solution around its surface. The results showed that this new material inhibited *S. mutans* biofilm growth and metabolic activity on its surface but had no effect on bacteria in solution. Even after 6 months of aging in water, the experimental material retained its antibacterial activity at a level similar to that observed without aging.

Bacterial infection is an important reason for failure of pulp capping procedures, especially in the treatment of caries [22], [33]. Previous studies have reported that the most common pulp capping materials used clinically, such as Dycal and MTA, lack sufficient antibacterial activity [10], [11]. Currently, studies on resin-modified pulp capping materials are popular. For example, Formosa *et al.* incorporated MTA into light- and chemical-cured resins to improve the ability of the resins to induce formation of mineralized tissue and to shorten the curing time of MTA [34]. Gandolfi *et al.* [35], [36] incorporated calcium-silicate PC-derived (MTA-like) fillers into light-curable resins to improve their mechanical properties and reduce the curing time of the MTA-like material, and their innovative materials were shown to promote the formation of bone-like carbonated-apatite on demineralized dentin. However, these novel materials showed no improvement in antibacterial activity due to the absence of an effective antimicrobial agent. Therefore, we attempted to apply a QAS antibacterial resin in the synthesis of a pulp capping material with antibacterial properties in addition to appropriate mechanical properties and the ability to induce mineralized tissue formation toward the goal of improving success rates for pulp capping treatment.

To achieve a material that promotes mineralized tissue formation, PC was incorporated into the novel material. We selected PC because it is the major component of MTA [24], [25] and also has been judged to have the same physical, chemical, and biological properties as MTA, such as alkalinization, calcium ion leaching, curing mechanism [26]–[28], biocompatibility [29], [30], and pulp capping effectiveness [22]. Moreover, studies using hydrophilic resins as direct pulp capping materials have reported promising results in animal models [37], [38]. Optimal compatibility of the QAS antibacterial resin with pulp tissue was also confirmed by direct pulp capping experiments in dogs [39]. Therefore, both PC and a QAS antibacterial hydrophilic resin to develop are reasonable choices for the design of a new pulp capping composite material.

Previous studies investigated antibacterial resins containing a QAS monomer that inhibits bacteria on contact, such as MDPB-containing materials and adhesives incorporating DMAE-CB [12], [13], [16], [19], [40], [41]. MAE-DB, a QAS monomer developed by our research group, has two reactive groups located on either end of the molecule. QAS monomers with two reactive groups have been shown to have little effect on the mechanical properties of the resin [31] and are expected to result in minimal monomer leaching, compared with other QAS monomers based on monomethacylates [19], [40]. The integration of MAE-DB in a resin matrix has also proved to be effective for providing chemically stable and long-lasting contact-inhibition of bacterial growth [20]. The antibacterial mechanism of MAE-DB involves the induction of bacterial lysis upon penetration and disruption of the cell membrane by the compound, which causes cytoplasmic leakage [21], [42]. When the negatively charged bacterial membrane contacts the positively charged (N^+^) sites of the QAS material, the electric balance of the cell membrane can be disturbed, and the bacterium may rupture as a result of the change in osmotic pressure [43]. Therefore, an antibacterial resin containing MAE-DB may offer a pulp capping material with antibacterial activity, and this hypothesis was confirmed by the results obtained in the present study.

The present study compared the antibacterial activity of the new pulp capping material with that of two currently commercially available pulp capping materials. The results for *S. mutans* growth and metabolic activity consistently demonstrated that the experimental material exhibited stronger antibacterial activity against *S. mutans* on its surface than did MTA and Dycal, whereas none of the materials affected bacterial growth or metabolic activity in the solution surrounding the samples. These findings are consistent with the contact-inhibition characteristic of QAS resins in general [17], [18], [21], [31]. The results of Live/Dead staining and SEM observation provide insights into the number and viability of bacteria on the material surfaces, and these results were consistent with those for metabolic activity and CFU formation. Interestingly, compared to bacterial behavior observed on the negative control material, the CFU-based results showed that MTA and Dycal displayed greatly increased the adherence of *S. mutans* biofilms on the surfaces by about an order of magnitude (*P*<0.05, Table 2). The results obtained via Live/Dead staining, SEM observation, and metabolic activity assays were all consistent with the CFU results. The increase in biofilm formation on these surfaces may be due to the release of calcium ions from MTA and Dycal upon reaction with phosphate ions in the BHI broth to produce calcium phosphate deposits on the material surfaces [36], which increases the surface roughness and thus promotes bacterial adhesion. Moreover, although CFU counts and metabolic activity results showed no significant differences between the experimental and positive control material surfaces regarding *S. mutans* biofilm formation, the results of Live/Dead staining and SEM observation showed that more bacteria adhered to the experimental material surfaces (and consequently exhibited loss of membrane integrity) than to the positive control surfaces. This may be because experimental material could also release calcium ions to produce calcium phosphate deposits on its surface in BHI broth, like MTA and Dycal can, and increased numbers of adherent bacteria were still killed by the antibacterial activity of the material.

The developed composite resin is cured by light irradiation for 60 s, and therefore, the curing time for the new composite material containing the light-curable resin is reduced to approximately 1 minute compared to 202 minutes for MTA [44], which improves the handling characteristics of the material. The curing time for the developed material is also somewhat shorter than that of Dycal (2.5–3.5 min) [45]. Although the pulp-capping effects of QAS antibacterial resin and PC have been confirmed by previous studies [29], [30], [39], that of the novel material combining both materials still remains to be verified in a further *in vivo* study. Furthermore, the present study only evaluated the antibacterial activity in an *in vitro* study, and additional important parameters for pulp capping materials such as the physical properties, chemical properties, bioactivity and biological properties, need to be evaluated in further experiments. Therefore, continued assessments of the physicochemical and biological properties of the material developed in this study are in progress.

## Conclusion

The results of the study indicate that, compared to commercial and negative control materials, the novel light-cured pulp capping composite containing PC as a filler and MAE-DB monomer as an antibacterial agent greatly inhibited *S. mutans* biofilm formation on its surface. Conversely, none of the tested materials had any effect on *S. mutans* growth in the culture medium surrounding the material samples. This contact-inhibition activity of the new pulp capping composite was shown to persist through 6 months of aging in water, suggesting that the developed material holds great promise for application as an antibacterial resin in pulp capping treatment.

## Supporting Information

Data S1 **Raw data.**
(XLS)
